# Medication eluting devices for the field of OBGYN (MEDOBGYN): 3D printed biodegradable hormone eluting constructs, a proof of concept study

**DOI:** 10.1371/journal.pone.0182929

**Published:** 2017-08-10

**Authors:** Karthik Tappa, Udayabhanu Jammalamadaka, David H. Ballard, Todd Bruno, Marissa R. Israel, Harika Vemula, J. Mark Meacham, David K. Mills, Pamela K Woodard, Jeffery A. Weisman

**Affiliations:** 1 Mallinckrodt Institute of Radiology, Washington University School of Medicine, St. Louis, Missouri, United States of America; 2 Department of Obstetrics and Gynecology, Louisiana State University Health Shreveport, Shreveport, Louisiana, United States of America; 3 Department of Pharmacy, St. Louis University Hospital, St. Louis, Missouri, United States of America; 4 Pharmaceutical Sciences and Chemistry, University of Missouri- Kansas City, Kansas City, Missouri, United States of America; 5 Department of Mechanical Engineering & Materials Science, Washington University, St Louis, Missouri, United States of America; 6 Department of Biomedical Engineering, Louisiana Tech University, Ruston, Louisiana, United States of America; 7 Department of Anesthesiology, Washington University School of Medicine, St Louis, Missouri, United States of America; Aristotle University of Thessaloniki, GREECE

## Abstract

3D printing has the potential to deliver personalized implants and devices for obstetric and gynecologic applications. The aim of this study is to engineer customizable and biodegradable 3D printed implant materials that can elute estrogen and/or progesterone. All 3D constructs were printed using polycaprolactone (PCL) biodegradable polymer laden with estrogen or progesterone and were subjected to hormone-release profile studies using ELISA kits. Material thermal properties were tested using thermogravimetric analysis and differential scanning calorimetry. The 3D printed constructs showed extended hormonal release over a one week period. Cytocompatibility and bioactivity were assessed using a luciferase assay. The hormone-laden 3D printed constructs demonstrated an increase in luciferase activity and without any deleterious effects. Thermal properties of the PCL and hormones showed degradation temperatures above that of the temperature used in the additive manufacturing process–suggesting that 3D printing can be achieved below the degradation temperatures of the hormones. Sample constructs in the shape of surgical meshes, subdermal rods, intrauterine devices and pessaries were designed and printed. 3D printing of estrogen and progesterone-eluting constructs was feasible in this proof of concept study. These custom designs have the potential to act as a form of personalized medicine for drug delivery and optimized fit based on patient-specific anatomy.

## Introduction

Major advances in biomedical engineering have led to new patient-specific technologies which treat diseases using biocompatible materials as controlled drug delivery systems. Additive manufacturing techniques, including Fused Deposition Modeling (FDM) 3D printing, boast high speed, accuracy, affordability, and feasibility leading to its increased use in medicine and biotechnology. Because of its rapid production and customizability, 3D printing has been used to construct prostheses, human tissues, medical devices, and surgical implants for dental and orthopedic applications [1–5].

Estrogens are available in various formulations and are administered via different routes such as intramuscular, subcutaneous, intravenous, transdermal, vaginal, and oral. Estrogen, progesterone, or combination formulations are variously used for contraception, regulation of the menstrual cycle, hormone replacement therapy (HRT), and vaginal atrophy among others. These drugs are delivered in a variety of forms. For example, extended steady-state release subcutaneous HRT implants are usually in the shape of a small pellet, made by fusing pure crystals of hormones, and are available at doses of 50–100 mg. Intrauterine devices (IUDs) are considered excellent long-term birth control because of their safety, efficacy, and ease of use [6]. These are small T-shaped devices, made of flexible plastic, are placed directly into the uterus, releasing small amounts of a progestin for up to 12 years [7]. Pessaries are medical devices used to treat pelvic organ prolapses by providing structural support to the uterus, vagina, bladder, and rectum [8], and are typically used in conjunction with local estrogen therapy. These devices are made in different sizes and designs that are subject to patient needs, anatomy, and physician preference [9].

Currently available obstetric and gynecologic implants are bulk manufactured with fixed doses, whereas every individual is unique, requiring different doses of hormones. Differences in IUD shapes and dosage concentrations leads to ectopic pregnancies, pelvic inflammatory disease, uterine perforations [10–11]. Commercially available IUDs cannot account for the differences between individuals. Bioactive 3D printing of IUDs and pessaries has the potential to provide patient-specific devices while delivering extended release hormones. The purpose of this study is to engineer customizable and biodegradable 3D printed implant material that can elute estrogen or progesterone.

## Materials and methods

### Materials

The Polycaprolactone (PCL) pellets used for filament extrusion were purchased from Sigma-Aldrich (St. Louis, MO). Hormones Estrone (E1) (SLE 1048), Estradiol (E2) (CAS 57-63-6) and Estriol (E3) (CAS 50-27-1), and Progesterone (P) (CAS 57-83-0) were purchased from PCCA, Houston, TX. Cell culture plates and other lab plastics were purchased from MidSci, St. Louis, MO. Rosewell Park Memorial Institute Medium (RPMI), Dulbecco’s Modified Eagle’s Medium (DMEM), fetal bovine serum (FBS), and penicillin-streptomycin-amphotericin (PSA) antibiotics were obtained from Life Technologies, Carlsbad, CA. Estrogen receptor luciferase reporter T47D stable cell line (Sl-0002-NP) was purchased from Signosis, Inc. (Santa Clara, CA). KJLC 705 silicone oil used for coating the beads before extrusion was purchased from Kurt J. Lesker Company (Jefferson Hills, PA). The 3D printing equipment consists of an ExtrusionBot extruder (ExtrusionBot, LLC Phoenix, AZ) and a MakerBot Replicator 5^th^ Generation Desktop 3D printer (Makerbot Industries Brooklyn, NY). The nanodrop spectrophotometer was from Thermo Scientific (Wilmington, DE). Solidworks 2015 student edition 3D computer aided design (CAD) program Dassault Systèmes (Waltham, MA) and Blender (Blender Foundation, Amsterdam, NL) were used for modeling. ELISA kits for E2 and E3 were purchased from Enzo Life Sciences (Farmingdale, NY). To analyze ELISA assays, a SpectraMax M2e Multimode microplate reader purchased from Molecular Devices (Sunnyvale, CA) was used. The SEM was a Hitachi S-4800 (Schaumburg, IL). Liquid chromatography- tandem mass spectrometry (LC-MS/MS) was performed on an AB Sciex 3200 OTrap mass spectrometer (Foster City, CA) interfaced with a Shimadzu UFLC HPLC system (Columbia, MD) using electrospray ionization (ESI) in positive mode, and run using Analyst® v.1.4.2 software. All chromatographic separations were performed on a Nucleodur 100–5 C8 125 x 3 mm column (Macherey–Nagel, Bethlehem, PA). Thermogravimetric analysis (TGA) and differential scanning calorimetry (DSC) were performed on a TA Q5000 IR and TA DSC 2500 (New Castle, DE) respectively. X-ray diffraction (XRD) was analyzed using Rigaku DMaxB instrument (Tokyo, JP).

### Fabricating hormone loaded scaffolds

PCL thermoplastic pellets were used for loading hormones due to its low melting temperatures, excellent biodegradability and biocompatibility. These pellets were coated with the calculated amount (1% w/w) of hormones and extruded as filaments of 1.75 mm diameter, which were used in Makerbot 1^st^ generation desktop 3D printer to print customized implant materials.

#### Coating pellets with hormones

**Fig 1:**
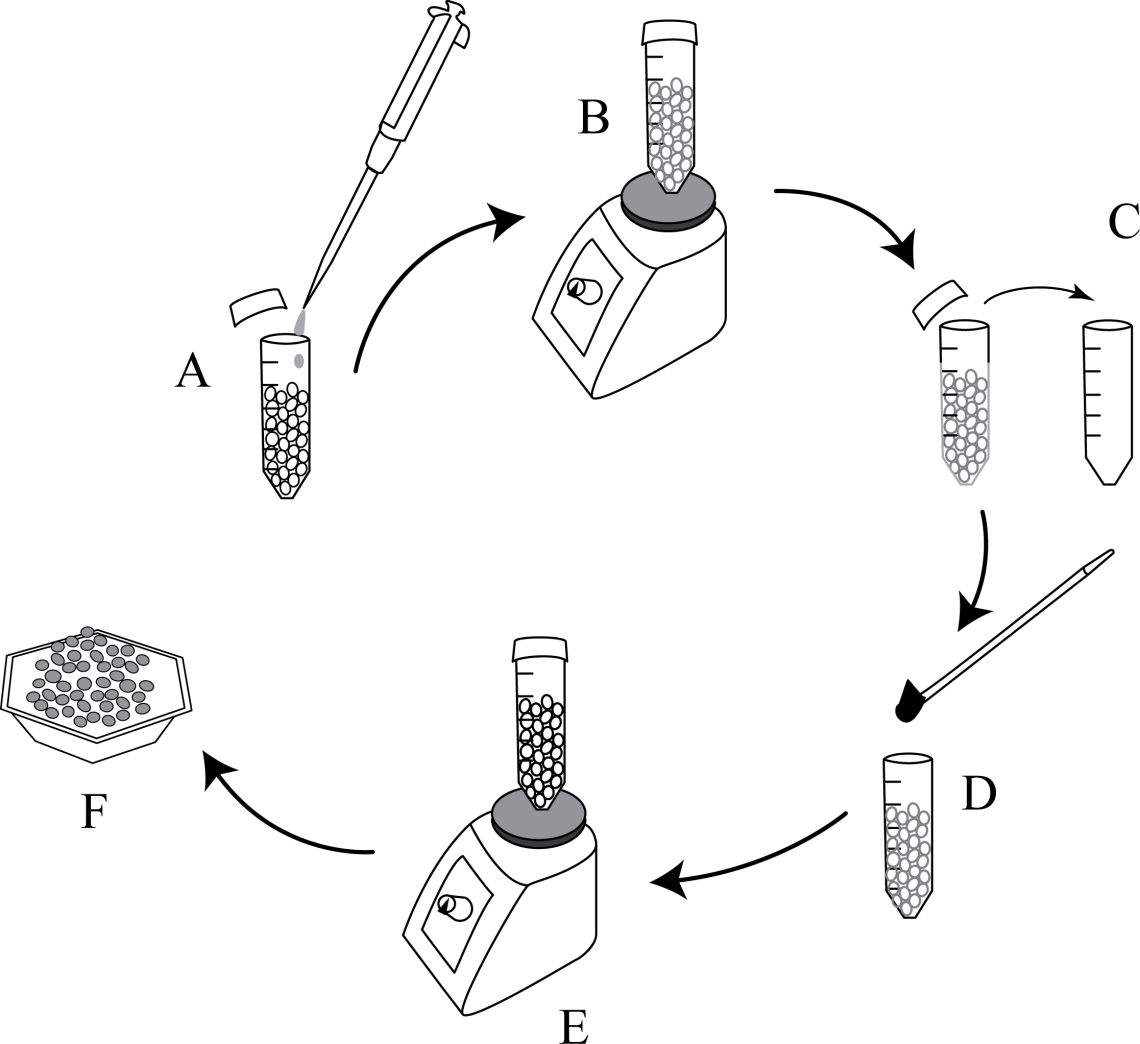
The process of coating PCL pellets with hormones. A) Coating oil is added, B) Tube is vortexed, C) Pellets are transferred to a new tube, D) Hormones are added, E) Tube is vortexed, F) Coated pellets are removed.

To enable an even dispersion of hormones on the surface of the commercially available PCL pellets, an oil coating method was used. To surface coat pellets, KJL 705 silicone oil was chosen because of its thermal stability at elevated temperatures. 15 μl of silicone oil was added to a 20 gm batch of pellets which were then vortexed to evenly and completely coat the pellets. Once vortexed, the pellets were transferred to another container to avoid loss of drug powder due to sticking to the surface of the oil coated mixing container. After switching containers, the calculated amount of hormones in powdered form was added and the mixture was vortexed again. This process is illustrated in **Fig 1**.

#### Extruding hormone loaded filaments

An ExtrusionBot filament extruder was used to extrude coated pellets. Since the 3D printer used requires a 1.75 mm diameter filament to print, a metal die of the same diameter was used to extrude the filament. Each 20 gm batches of E1, E2, E3, and progesterone were extruded at 90–100°C.

#### Optimizing 3D printing parameters

3D printer based on FDM is a very delicately balanced machine. If filament-extrusion temperature, the speed of extrusion and speed of the printer-head are not coordinated, the printing process does not yield constructs of the required shape. An optimized process also involves complex integration between hardware, software and material properties. Primary trials revealed that the printer-head temperature and filament feed rate have a direct influence on the material flow for the fabrication of the scaffolds. For this reason, optimization of printing parameters for PCL was focused mostly on printer-head temperature and filament feed rate. The minimum temperature of the printer-head that could print PCL was determined, and the filament extruding rate was adjusted accordingly.

On the MakerBot 1^st^ generation 3D printer, PCL prints at a lowest temperature of 100°C, and to compensate for this, the printer-head speed was reduced from a default 40 mm/s to 10 mm/s and increase the filament feed rate from a default 18 mm/s to 23 mm/s. All E1, E2, E3 and progesterone constructs were printed at 110°C at a printer-head speed of 10 mm/s and a constant filament-feed rate of 23 mm/s.

### Scaffolds design and fabrication

All 3D models including IUDs, meshes, and pessaries were designed using Solidworks 2015 CAD software. Discs of 5 x 1 mm dimensions were printed for drug elution testing and *in vitro* cell cultures. IUDs and pessaries of different dimensions and shapes were also designed and printed using the extruded filaments.

### Scaffold characterization

The 3D printed hormonal scaffolds were subjected to physical and bioactive testing. Surface morphology of the constructs was studied using SEM. Thermal properties were evaluated using TGA and DSC. Crystalline and amorphous states of the constructs were analyzed using XRD. ELISA kits were used to study hormonal release from scaffolds, and Luciferase assay was conducted to test the bioactivity of the hormonal discs.

#### Morphology

Extruded filaments and printed discs of different hormones were subjected to SEM using S4800 Field Emission SEM, HITACHI (Schaumburg, IL) at different magnifications. Construct surface were coated with a thin film of gold before imaging to make them conductive.

#### Thermal properties

Given that polymers undergo heating during 3D printing, thermal degradation behavior of polymer, hormones and the 3D printed constructs were studied using TA Instrument Q5000 IR. The samples were tested using ramp method. Samples were heated from ambient temperature to 500°C at a rate of 20°C/min and changes in sample weight were measured. Changes in melting temperatures (T_*m*_) and glass transition temperatures (T_*g*_) of polymers due to addition of hormones were tested using DSC. Samples were heated from -80°C to 130°C at a rate of 10°C/min. Energy input (heat flow) was measured as a function of temperature in this analysis. Both analyses were carried out in a nitrogen environment.

#### X-ray diffraction

Crystalline and amorphous properties were analyzed using XRD. Rigaku DMaxB instrument was operated at 35K and 30mA to produce radiation from Copper. Diffractions were measured within the range of scattering angles (2θ) of 10° to 50° with dwell time of 2 min.

#### Absolute quantification of hormones using tandem MS/MS analysis

An LC-MS/MS method was developed for the separation and quantification of different hormones in the scaffolds using mass spectrometry. A gradient elution method was employed with solvent A (HPLC water and 0.1% formic acid) and solvent C (acetonitrile and 0.09% formic acid) as the mobile phase solvents with a total run time of 14 min and flow rate of 0.3 mL/min. The elution profile starts with 10% solvent C ramped to 60% in 8 min and 100% in next 2 min, followed by post-equilibration with solvent C to 10% for 4 min. The UV detector was set at 280 nm. A multiple reaction monitoring method was developed with 1mM standard solutions of E1, E2, E3 using the signature transitions for each analyte- E1, E3 (271.3/133.0) and E2 (255.0/159.0). Method development included validation for accuracy, specificity, sensitivity, determination of response linearity, limit of detection (LOD), lower limit of quantification (LLOQ) and possible matrix effects and carryover.

Samples were prepared by suspension and extraction of the discs in diethyl ether. The dried extract is resuspended in 50 uL of solvent A and analyzed by LC-MS.

#### Hormone elution profile

To calculate the amount of hormone eluting from the constructs, an elution study was conducted. Saline was used for sample collection. Samples from E2 and E3 were collected at periodic intervals for a week. These collected samples were analyzed using appropriate ELISA kits. From the ELISA pilot studies, it was determined that the concentration of E2 eluting from the scaffolds was above the maximum detection level and all measurements were near saturation. A ten-fold dilution of the E2 samples and ELISA was performed. E3 samples had absorbance values within the ELISA detection range and were not diluted. The SpectraMax M2e multimode microplate reader was used to read the 96 well plates absorbance values. From the absorbance values of standards, the four parameter logistic (4PL) nonlinear regression model was drawn, and concentrations of unknown samples were back calculated. For all the assays, the absorbance values at 405 nm were measured and recorded.

#### Hormonal activity

The hormonal activity of E2 in the 3D printed scaffolds was assessed as follows. Plain PCL pellets, saline and different concentrations of E2 were used as controls. Extruded filaments and 3D printed discs were used as unknowns. Before these scaffolds were seeded, 90% confluent endometrial cells were detached from the culture tubes and suspended in RPMI medium. Each well was filled with 10 μl of suspension and incubated for 24 hours for attachment. Scaffolds were added to these wells and incubated for another 16 hours. Lysis buffer was added to each well and incubated for two minutes. In a new 96 well plate, 20 μl of this lysate was transferred, and 100 μl of luciferase substrate is added. Optical density of each well was immediately read using a luminometer.

### Statistical analysis

For the ELISA assay, three samples of each batch were tested and averaged for reproducibility. Standard deviations of the means were calculated and mentioned in the elution profile graphs as error bars. For the *in-vitro* study, measurements from five wells of each group were averaged. A one-way ANOVA was conducted to analyze the significant difference between the means among the groups. The standard deviation of the means was calculated and represented as error bars in the graphs.

## Results

**Fig 2:**
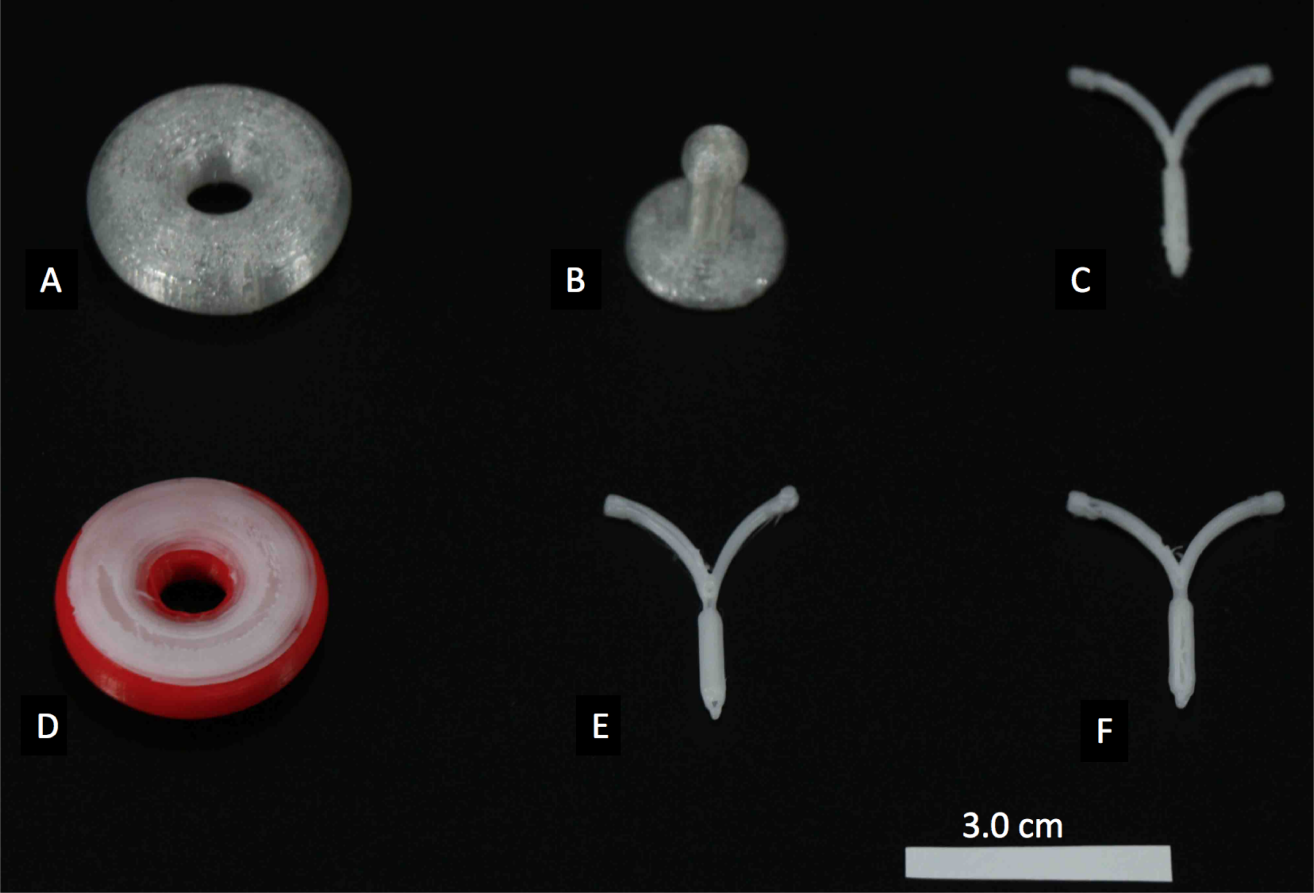
**3D printed constructs** A) Control donut shaped pessary, B) Control Gellhorn shaped pessary, C) Control IUD, D) Pessary printed combinations of filaments (red- PLA and white- PCL-E2), E) PCL-E1 IUD, and F) PCL-E2 IUD.

**Fig 3:**
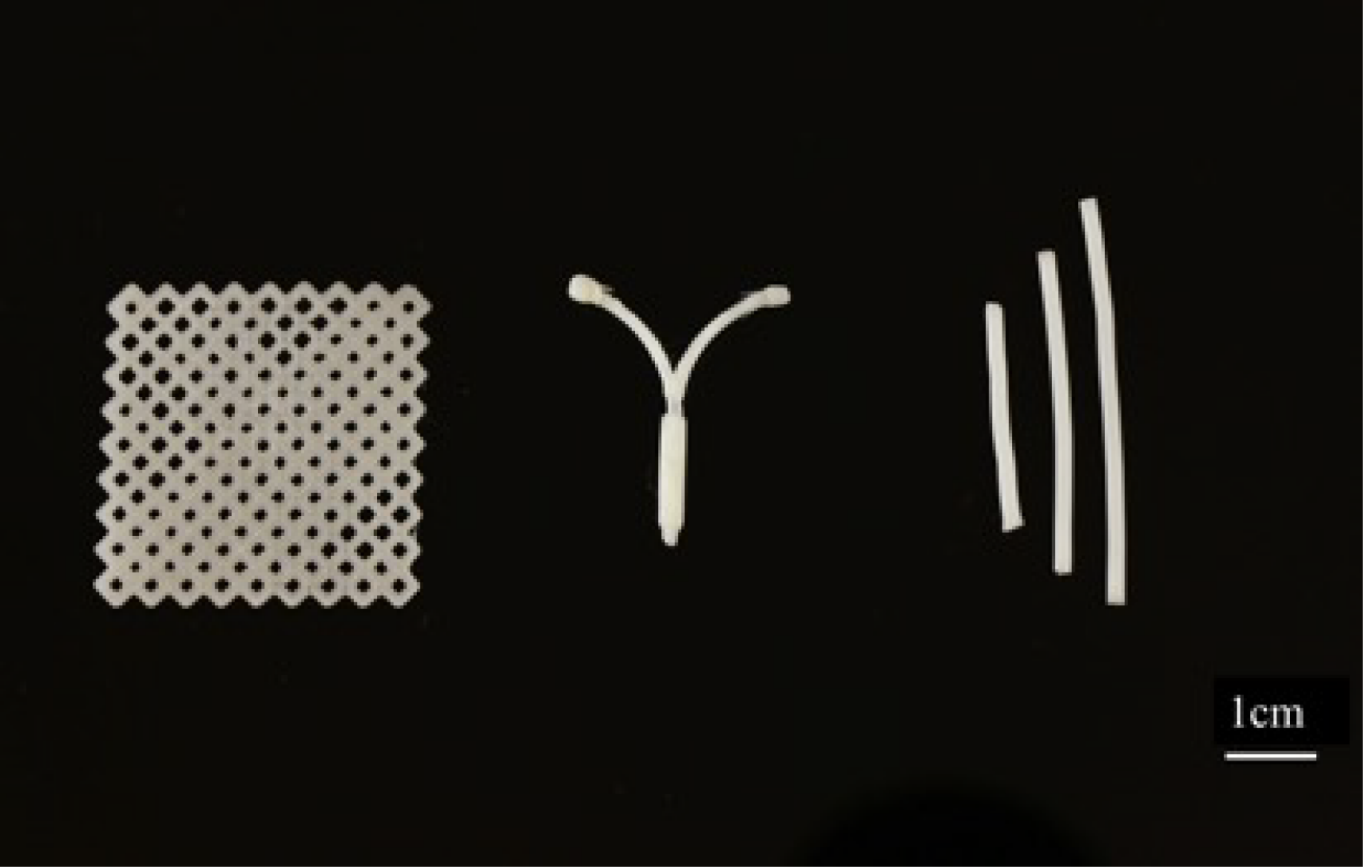
3D printed PCL-Estrogen mesh, PCL-Progesterone IUD, and Subdermal implant.

PCL pellets were successfully coated with E1, E2, E3 and progesterone hormones. Each 20 gm batch of 1.75 mm diameter filaments were extruded, and the required constructs were successfully printed. **Fig 2**shows images of PCL constructs printed with and without hormones using the Makerbot 3D printer. Constructs containing a combination of hormones can be printed using this technique. **Fig 2D** shows a donut shaped pessary printed with unloaded polylactic acid (PLA) filament (red) and with PCL filament (white) loaded with E2 hormone. **Fig 3**shows of surgical mesh printed using PCL-Estrogen and an IUD, rod shaped implant 3D printed using Progesterone loaded PCL filaments.

### Scanning electron microscopy (SEM)

**Fig 4:**
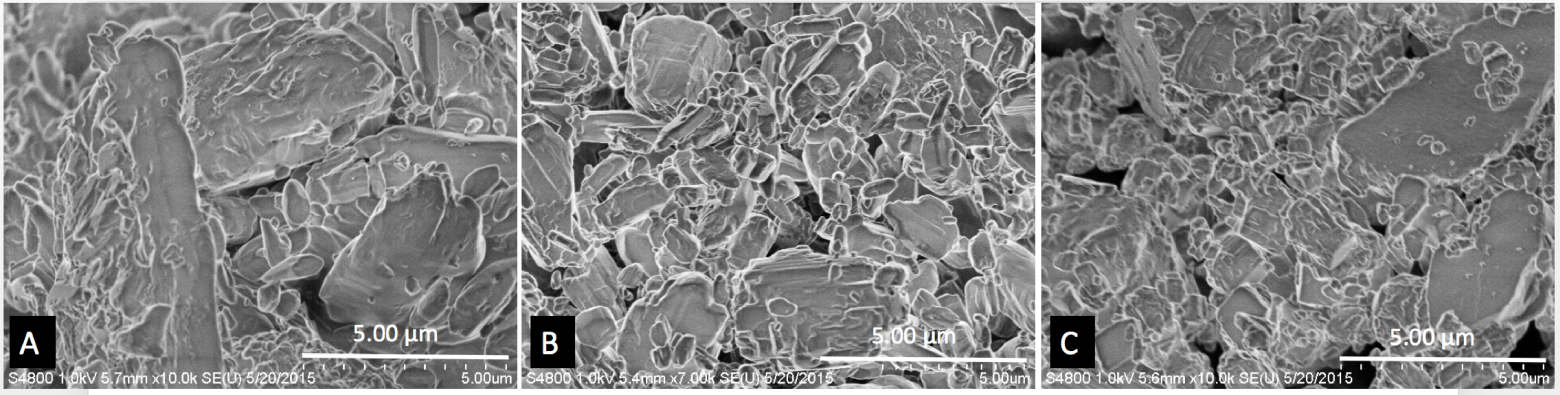
SEM images of hormones. A) E1, B) E2, C) E3.

SEM was used to study the surface morphology of the coated pellets, filaments, and printed discs. **Fig 4**shows the SEM images of estrogen hormones E1, E2, and E3.

**Fig 5:**
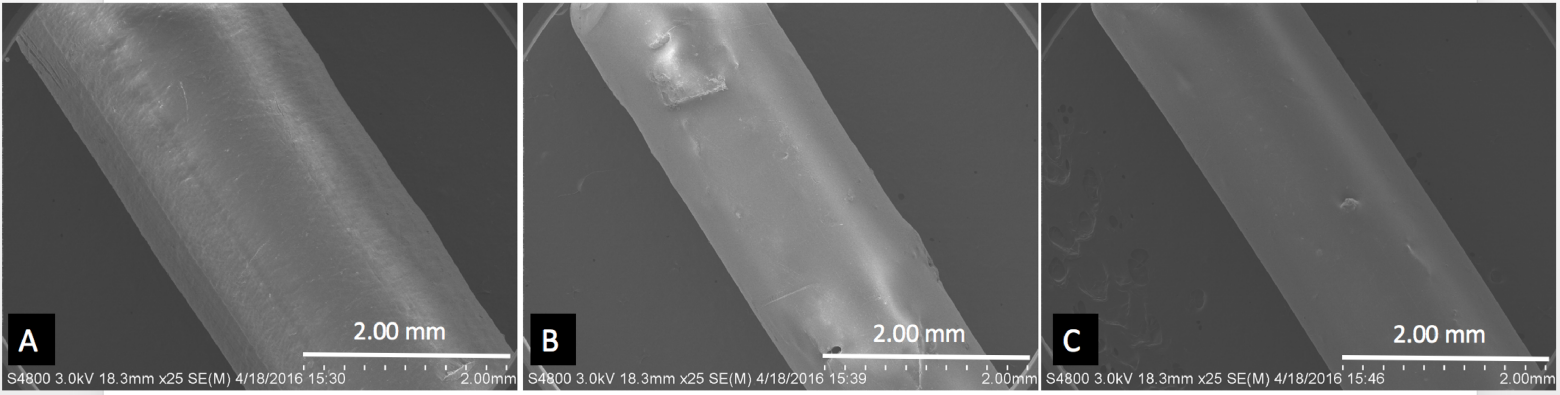
SEM images of filaments. A) E1, B) E2, C) E3.

All these hormones contain irregularly shaped particles of varied sizes. The estimated largest size of these particles was around 5 μm. Filaments extruded from ExtrusionBot were cylindrical in shape and had a smooth external surface. The SEM image in **Fig 5**shows hormone loaded PCL filaments extruded using the ExtrusionBot filament extruder.

**Fig 6:**
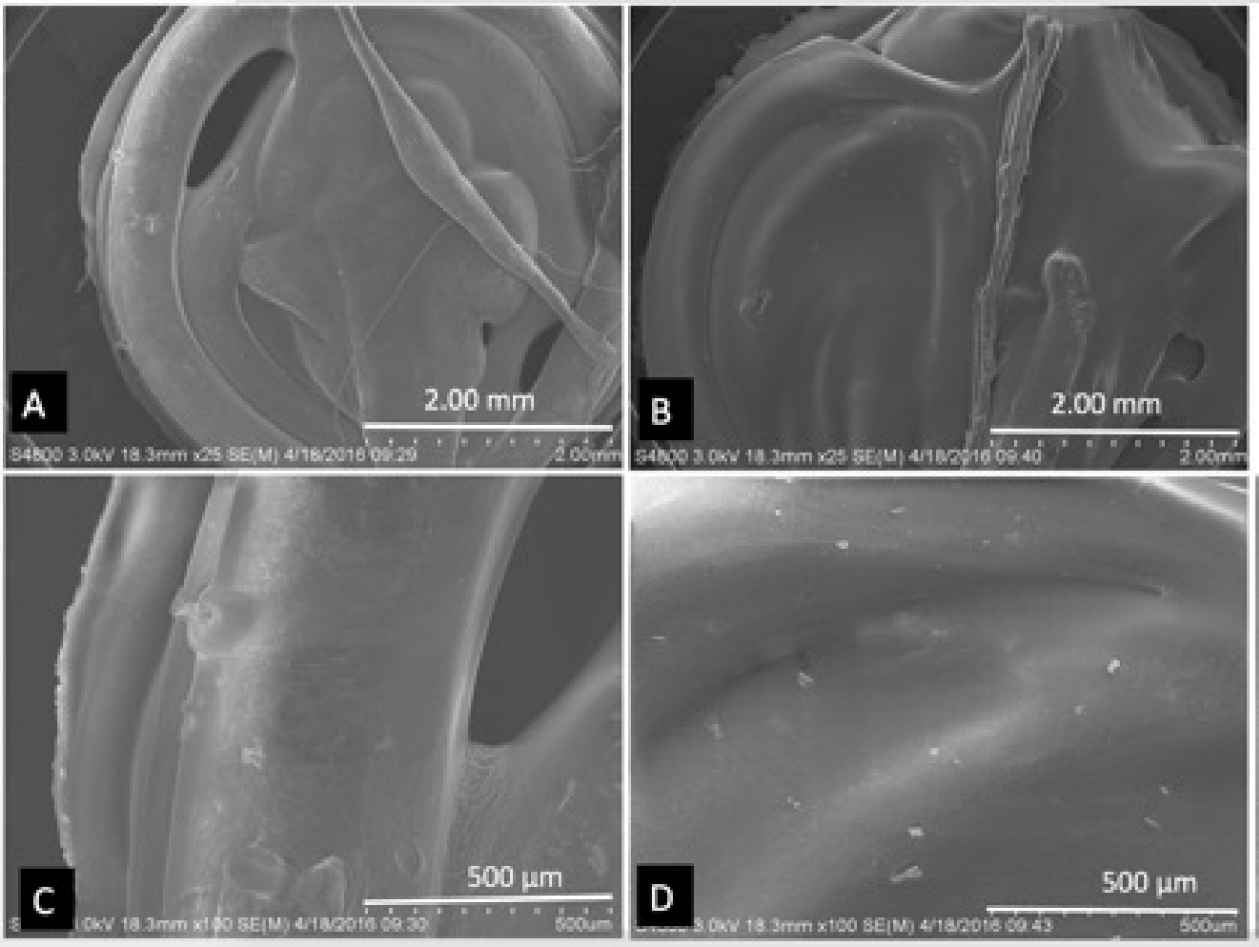
SEM images of discs. (A, C) PCL-E1 Discs, (B, D) PCL-E2 Discs.

**Fig 7:**
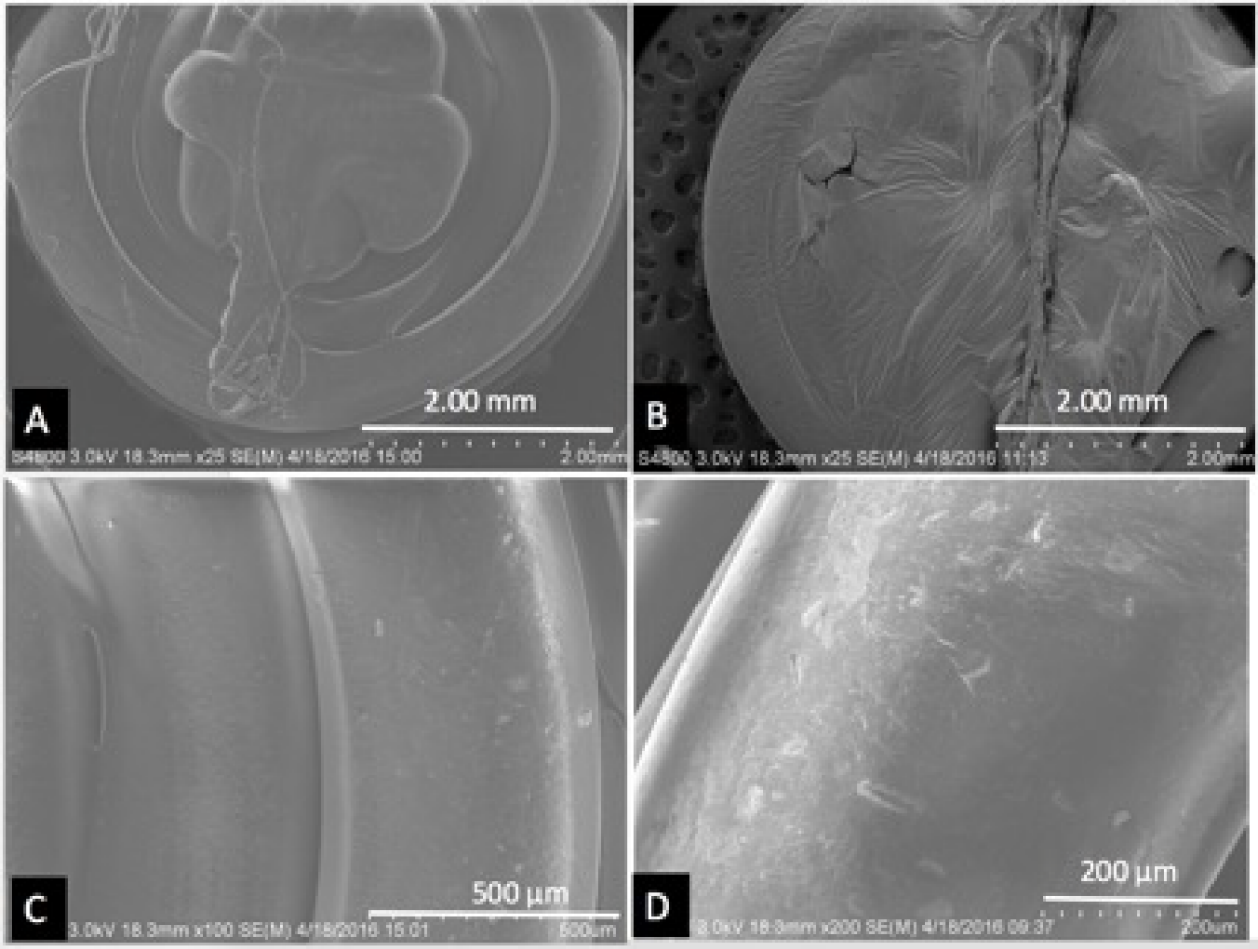
SEM images of discs. (A, C) PCL-E3 Discs, (B, D) PCL-Progesterone Discs.

Extruded filaments were used in Makerbot 3D printer to print discs of 5 mm × 1 mm dimensions. **Figs 6**, **7A and 7B** shows all 3D printed discs used for ELISA and cell culture at 25X magnification. These images exhibit layers of filaments comprising cylindrical shape with 300 μm in diameter and the sintering between the layers. On further magnification, hormone particles were seen on the surfaces of these discs. **Figs 6, 7C and 7D** shows E1, E2, E3 and Progesterone particles on the surfaces of the discs.

### Thermal properties

**Fig 8:**
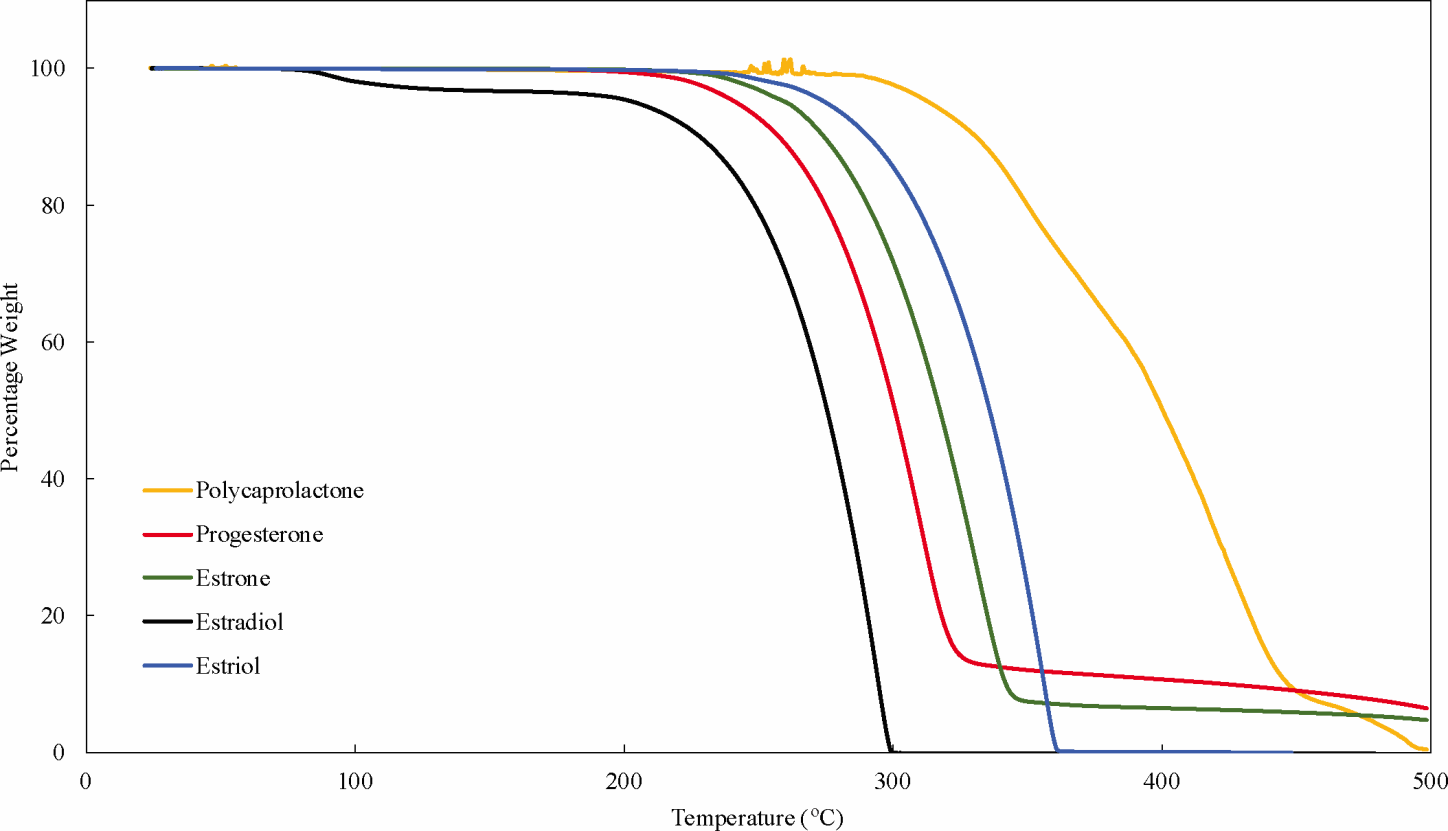
TGA thermograms of PCL and hormones.

All the hormones started thermal degradation at 190–240°C and PCL degradation was observed to start at 280°C (**Fig 8**). The estradiol thermogram shows a 4% reduction in weight at 100°C suggesting the presence of residual moisture in the estradiol sample.

**Fig 9:**
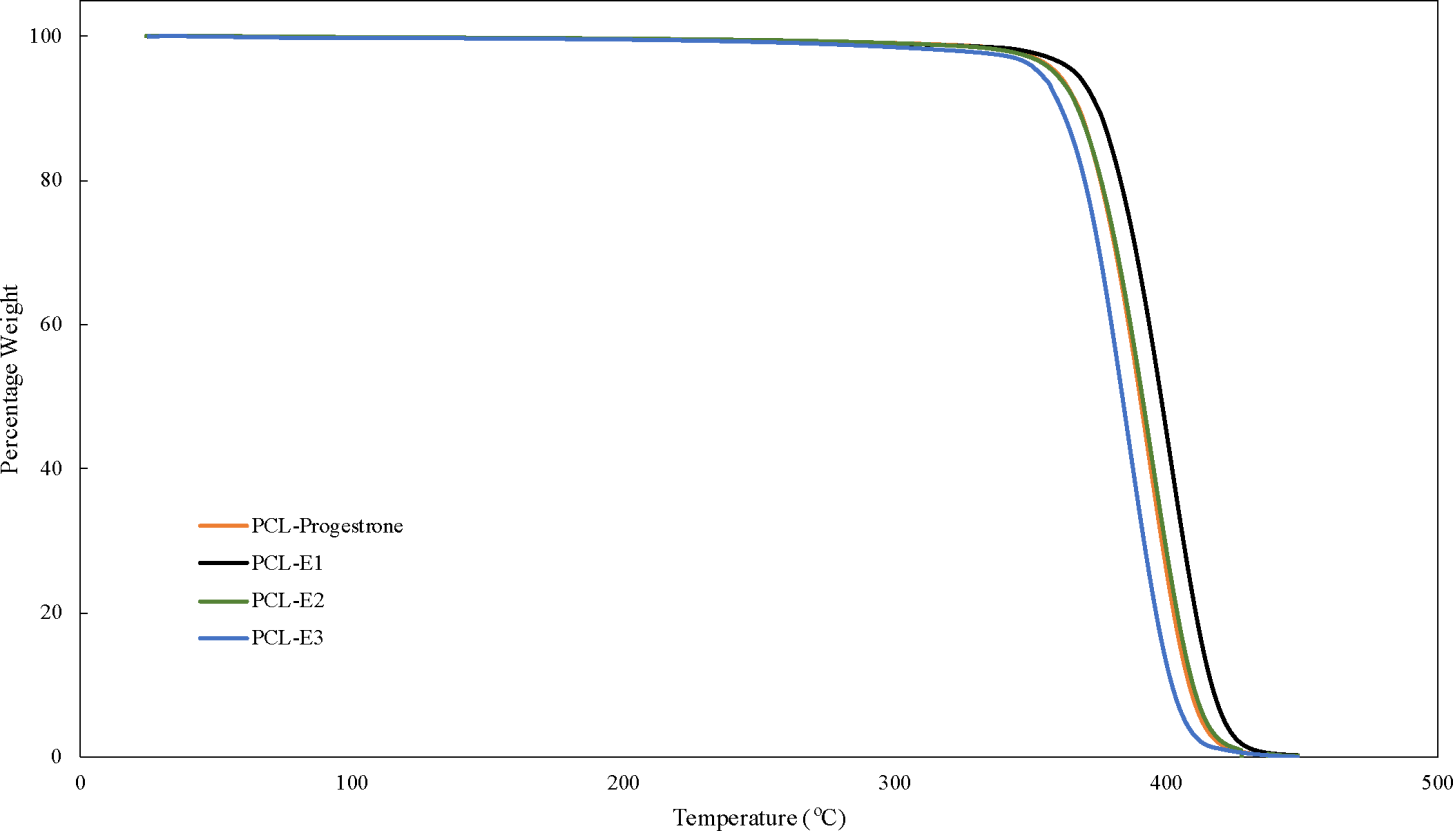
TGA thermograms of PCL-hormones composites.

While the hormones initially had degradation temperatures ranging from 190–240°C, incorporating them in PCL matrix rendered both hormones and PCL more thermally stable. Thermal degradation of all the 3D printed composites containing PCL matrix and hormones had degradation starting at 310°C (**Fig 9)**.

**Fig 10:**
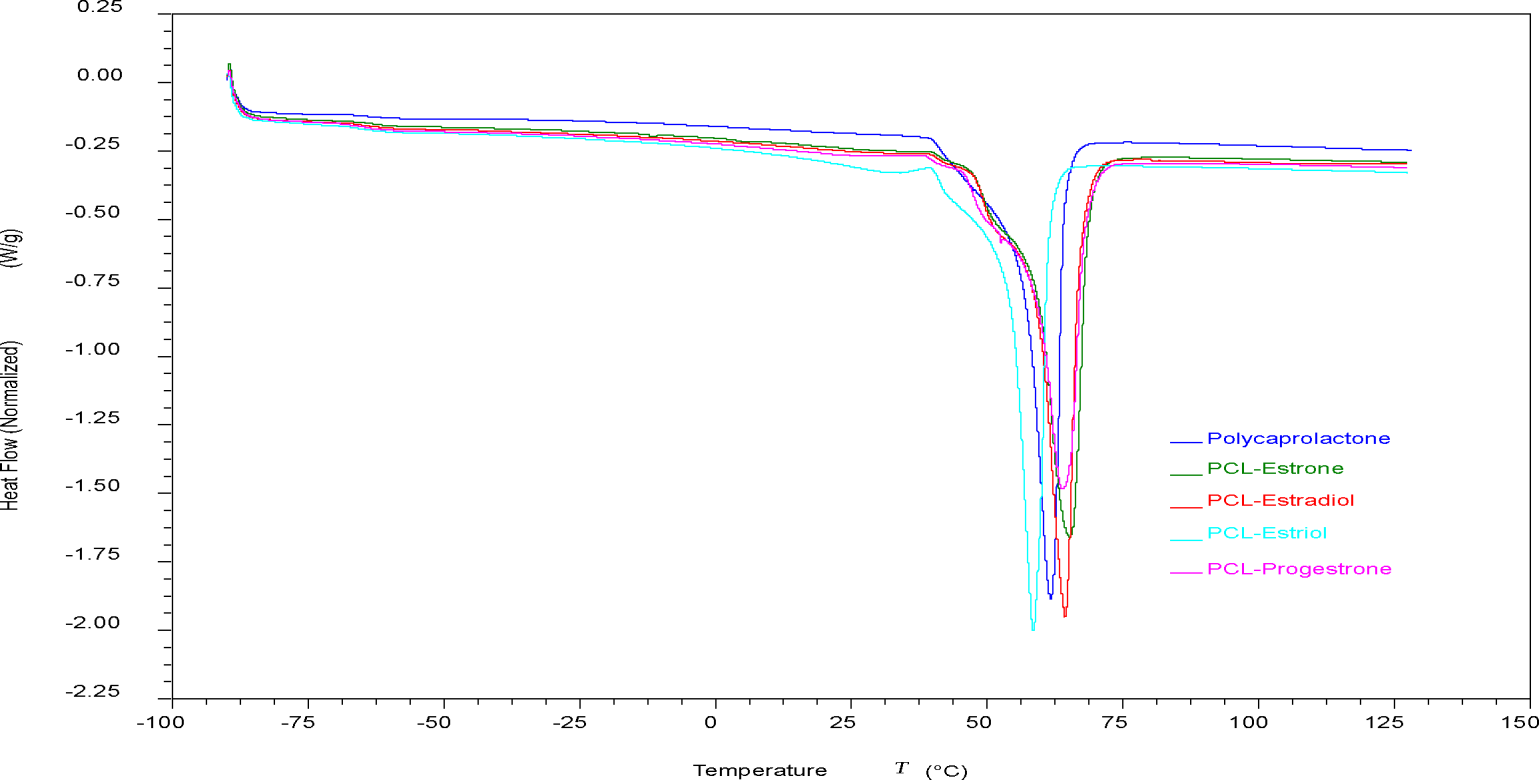
DSC thermogram of PCL-hormone composites.

Differential scanning calorimetry results suggest the changes in T_*g*_ and T_*m*_ of the polymer composites was not pronounced as shown in **Fig 10**. Small changes in T_*g*_ were observed in the composites when compared with PCL. Glass transition temperature of PCL was observed to be at -62.8°C while the composites’ T_*g*_ ranged from -64.9°C to -63.3°C. For PCL, T_*m*_ was observed to be 61.7°C and the composites had melting temperatures ranging from 65.2°C to 58.5°C.

### X-ray diffraction

**Fig 11:**
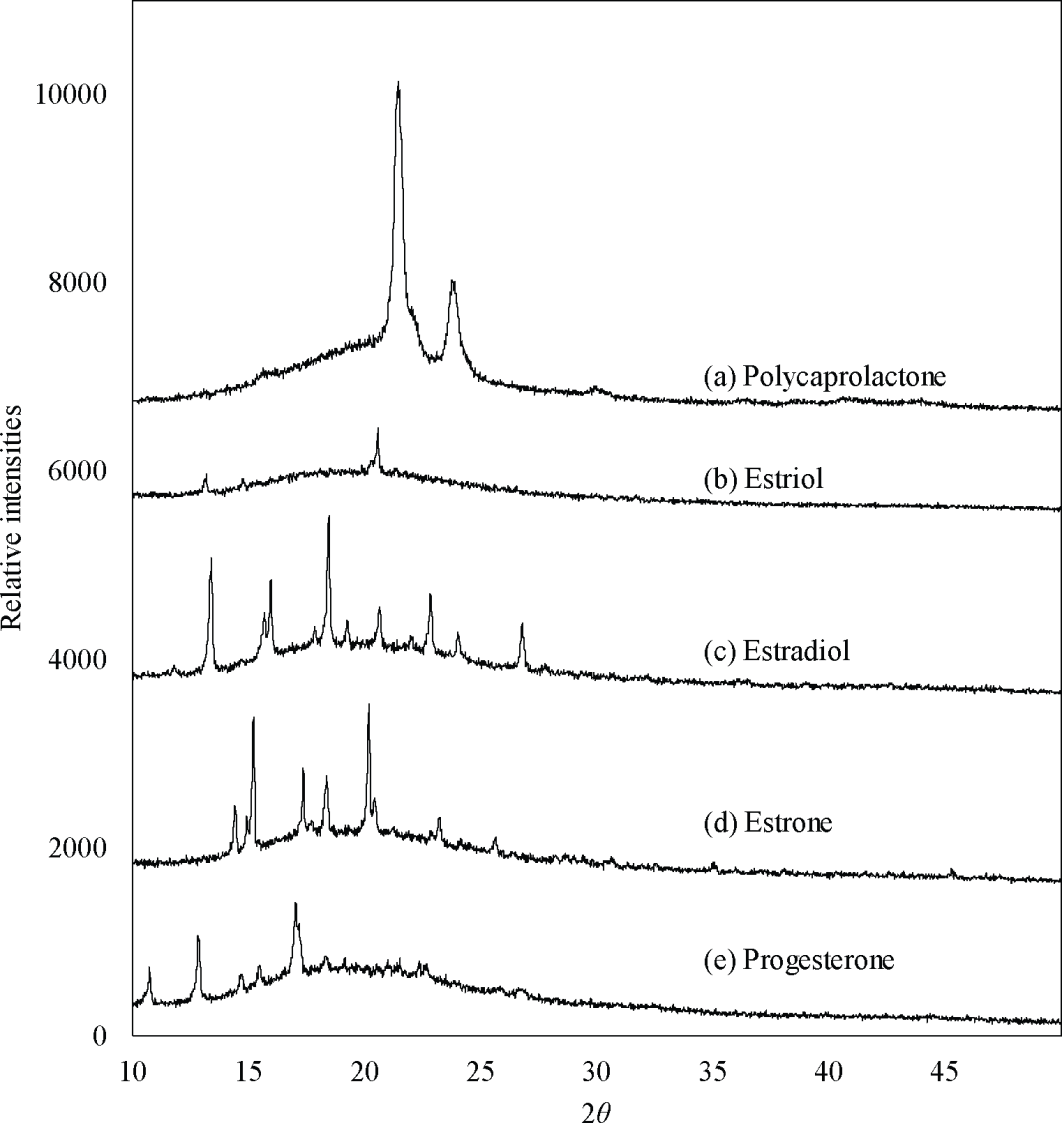
XRD diffractograms of polymer and hormones.

**Fig 12:**
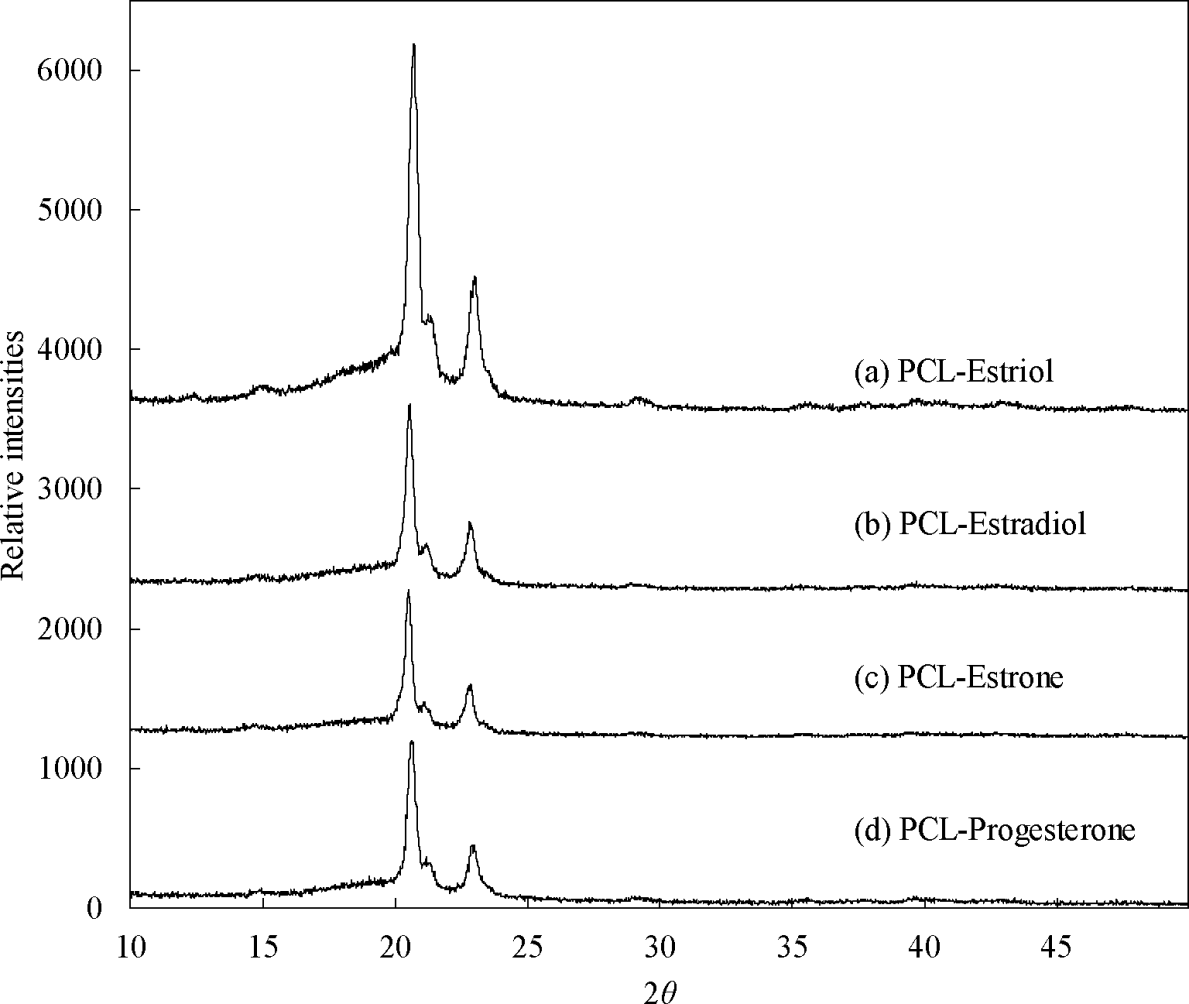
XRD diffractograms of PCL-hormones composites.

Diffractograms of PCL and hormones show their amorphous and crystalline phases (**Fig 11**). Crystalline phases in PCL are attributed to the strong peaks at 21.4° and 23.8°. Amorphous scatter of PCL is observed over a range of 14° to 25°. Crystalline scatter of estriol is seen at 13.2° and 20.5°. Multiple crystalline scatter peaks were observed for estradiol at 13.4°, 15.9°, 18.4°, 20.6°, 22.8°, and 26.8°. Peaks observed at 14.5°, 15.2°, 17.4°, 18.4°, and 20.2° for estrone are due to the crystalline phase in the powder. Crystalline scatter peaks for progesterone were observed at 10.7°, 12.8°, and 17°. For all the hormones, amorphous scatter was seen as a large bump at 2*θ* ranging from 10° to 30°. Diffractograms of PCL-hormone composites are shown in **Fig 12**. All the diffractograms had the same pattern as PCL polymer.

### LC-MS/MS detection of hormones from implants

Using LC-MS, successful detection, separation and absolute quantification of different hormones (E1, E2, E3, and Progesterone) were performed, given the same transitions (271.3/133.0) for E1 and E3 (**S1 Fig).** The elution of hormones from the column is based on the hydrophilicity of analyte with E3 eluting first followed by E2 and E1. The results are further confirmed by comparing these findings to their respective peaks in UV chromatogram at 260nm. The concentrations of different hormones in the constructs were also determined with a control (negative) where no analytes were detected (**S2 Fig**).

### Quantification through ELISA

Preliminary studies showed the release of hormones were in nanograms concentration. A ten-fold dilution of the samples was done to meet the ELISA kits detection range. Samples were collected, one for each day. For the first two days, 35% of total hormone was released from the scaffolds.

**Fig 13:**
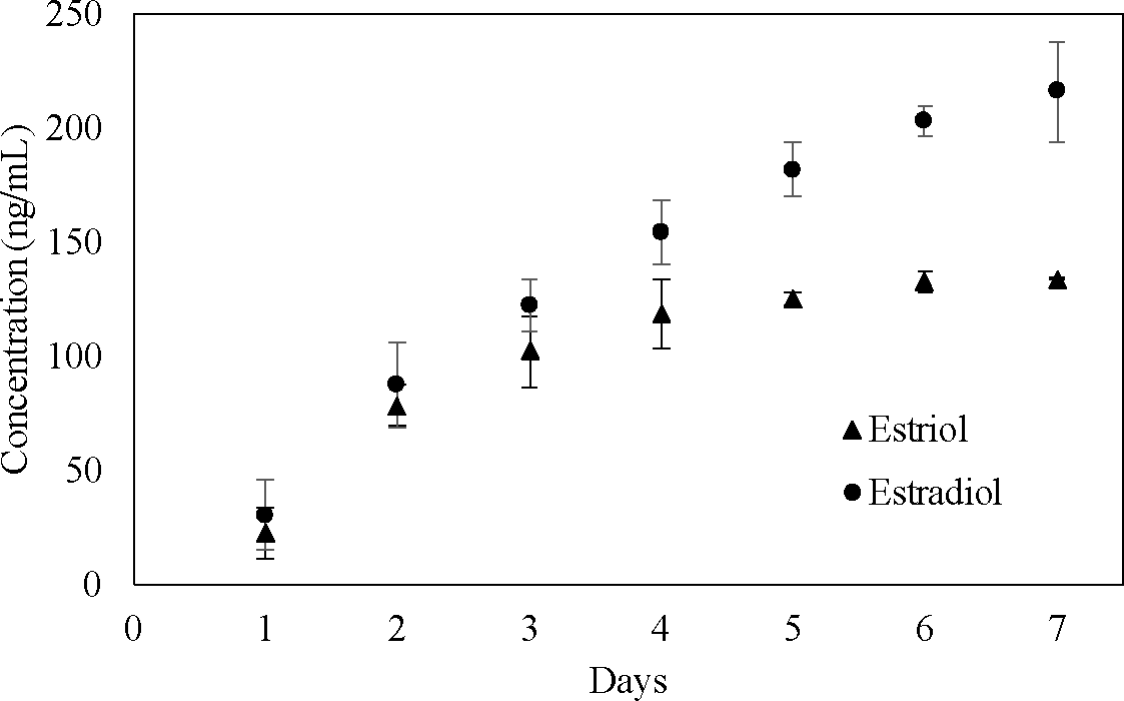
Cumulative concentration of E2 and E3 released from PCLcomposite discs (mean ± SD, n = 3).

**Fig 13**shows the concentration of hormone E2 released over a period of one week. A total of 215 ng/mL of the cumulative concentration of E2 hormone was released at the end of day seven. Even after seven days, small quantities of hormone release within the working concentration (2 ng/mL -5 ng/mL) were measured.

All the hormone loaded discs were 3D printed at 300 μm resolution. Preliminary tests showed the release of E3 hormones from the constructs were within the detectable range of the ELISA kits. For the first two days, 46% of total E3 hormone was released from the scaffolds. On day seven, 1.3 ng/mL hormone was released.

**Fig 13**also shows the cumulative concentration of E3 released from PCL scaffolds for seven days. A standard graph plotted from the known concentration was used to yield a four parameter logistic curve. E3 release increased during the first four days, after which hormonal elution was steady and extended. A total of 118 ng/ml (cumulative concentration) of E3 hormone was released by the end of day four.

### In vitro assay

**Fig 14:**
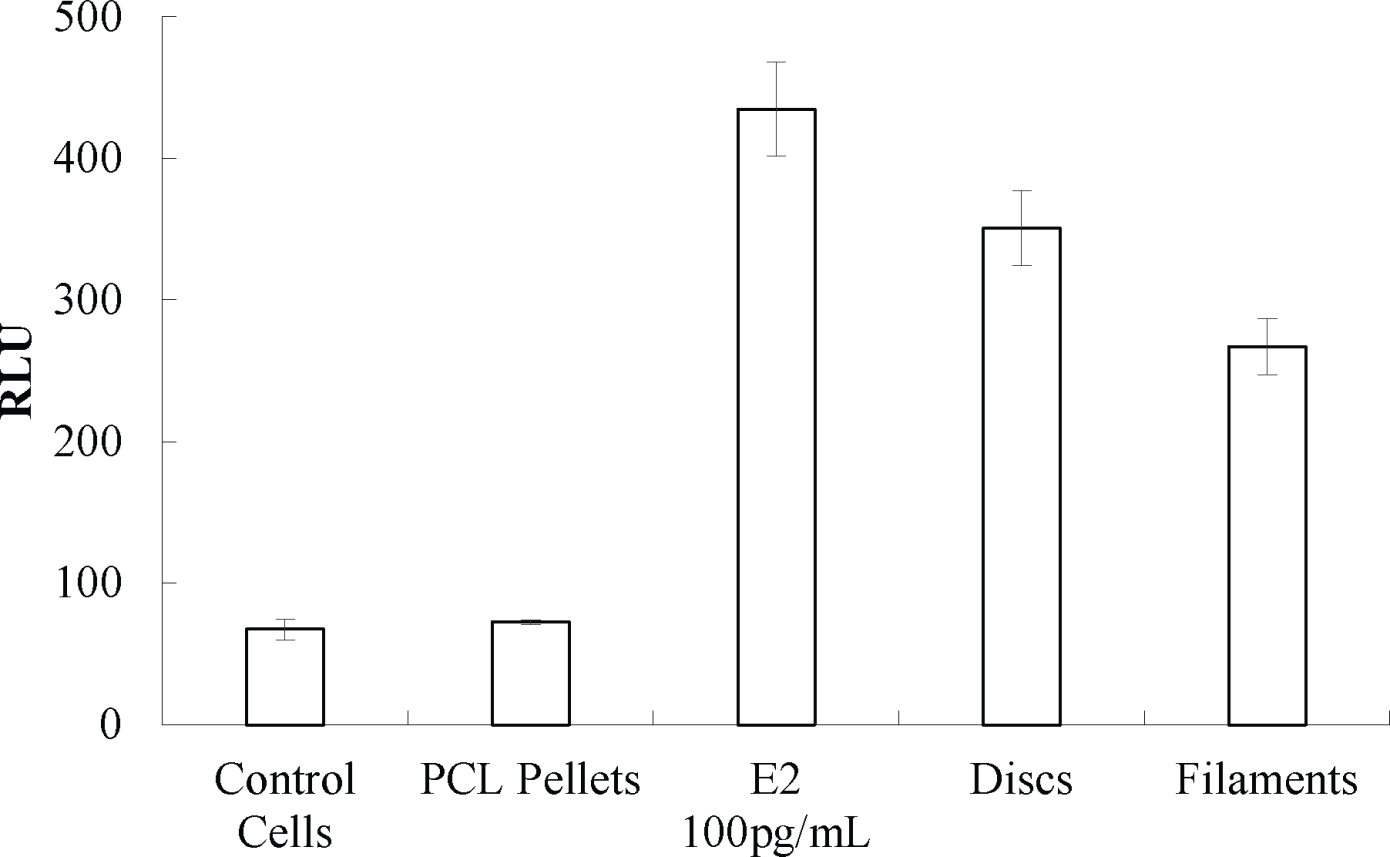
RLU values of Estrogen receptor luciferase reporter cells incubated with different groups of E2 scaffolds (mean ± SD, n = 5).

**Fig 14**shows the Relative Light Unit (RLU) values of luciferase activity in response to estrogen stimuli. Five wells of each group were tested and ANOVA was conducted. The difference between the control cells and control PCL pellets was not statistically significant (*p* ≤ 0.05), indicating that the PCL pellets are biocompatible and do not promote any estrogen induced pathways. The group containing E2 showed a robust increase in RLU values when compared to groups without inducing agent, indicating the presence of bioactive compounds in the fabricated constructs. Wells containing 100 pg/ml concentration of E2 showed a total of 84.6% more luciferase activity. Luciferase activity for extruded filaments (1 cm length) and 3D printed discs (5 mm X 1mm) increased by 80.85% and 74.9%, respectively, when compared to the control cells activity.

## Discussion

Fused deposition modeling was used to fabricate hormone loaded biodegradable implant materials. PCL pellets were coated with estrogen (E1, E2, E3) and progesterone, then filaments of required dimensions were successfully extruded. With these bioactive enhanced filaments, constructs of relevant designs were fabricated using 3D printing. SEM images showed extruded filaments were smooth and cylindrical in nature. Thermal properties of the PCL, hormones and composites indicate that 3D printing can be achieved well below their degradation temperatures. Given that thermal degradation of both the hormones and PCL polymer began at 190°C or greater and 3D printing of all the constructs was done at 130°C, it can be inferred that both hormones and polymer were not thermally degraded during the 3D printing process. Additionally, the transitional temperatures of the 3D printed constructs were not greatly affected due to the presence of hormones. The absence of amorphous scatter (other than PCL) in the diffractograms may signify that changes in crystalline phases of hormones did not occur. As the composites had low concentration of 1% w/w, it is possible that the x-rays could not interact with hormones to cause any diffraction or the heating during the 3D printing process may have affected their crystalline phases.

The chromatographic peaks for hormones E1, E2, E3, and progesterone show the presence of bioactive compounds in 3D printed constructs. The study of release kinetics using ELISA assay showed that the hormones E2 and E3 were released at a steady rate from the scaffolds over a short period. *In vitro* assays conducted on estrogen receptor luciferase reporter cell lines show that the fabricated scaffolds were biocompatible. Groups enhanced with hormones showed robust luciferase activity, indicating the presence of a bioactive inducing substance (E2) in the scaffolds.

Bioactive 3D printing has been the focus of several in vitro investigations to deliver antibiotics and chemotherapeutics. Weisman et al. [12] used fused depositional modeling to create 3D-printed gentamicin- and methotrexate-laden constructs. The drug-laden bioactive constructs showed antimicrobial and tumor-inhibiting effects, both with favorable elution profiles. In another study, this concept was implemented in antibiotic and chemotherapeutic impregnated catheters and showed similar in vitro results [13]. Ballard et al. [14] used fused deposition modeling to create customized surgical meshes. In that study, gentamicin-laden meshes inhibited bacterial growth compared to none by control 3D-printed surgical meshes.

There have been few studies that use 3D printing for implications in obstetrics and gynecology. Tudela et al. [15] used prenatal ultrasound measurements of cervical length and radius to fabricate custom 3D printed cerclage pessaries. Hakim et al. [16] fabricated vaginal stents and dilators of customizable sizes and shapes from computed tomographic-acquired data from commercial products. In radiation oncology, 3D printing has been used in a small number of patients to fabricate custom vaginal cylinders for vaginal brachytherapy [17–19]. Sethi et al. [19] reported 3D printing custom vaginal applicators for brachytherapy in patients with unique and postsurgical vaginal anatomy. Finally, the creation of bioprosthetic ovaries in mice has been facilitated using 3D printing. Laronda et al. [20] 3D printed porous gelatin scaffolds with stiffness to mimic ovarian tissue (based on reference standards) and pore size and morphology to accommodate ovarian follicles. After substantiating their finding *in vitro*, they implanted the 3D printed ovarian-like implants into mice with oophorectomies. These scaffolds were populated with ovarian follicles in various stages of maturity. Over time, the development of these bioprosthetic ovaries demonstrated folliculogenesis and development of blood vessels throughout the scaffolds. Further, three of seven mice with 3D printed bioprosthetic ovaries demonstrated fertility and bore offspring [20].

The present study offers the first data to show hormone-laden 3D printed constructs. While the potential benefits, pitfalls, and clinical correlates for the estrogen and/or progesterone-eluting constructs fabricated in this study are unclear, the ability to impregnate 3D printed constructs with bioactive hormones portends new possibilities in additive manufacturing of implants and devices.

The resolution of the 3D printed constructs can be changed by altering the layer height of the deposited filament. With decrease in layer height of the filament, surface area of the construct increases and should, in turn, increases the hormonal release.

The ability to 3D print estrogen and/or progesterone-loaded biodegradable implants holds great potential in personalized medicine. Using these methods, IUDs, and pessaries could be tailored to a patient’s specific needs in aspects of the unique anatomy of the individual, hormone dosage and period of hormone therapy.

## Supporting information

S1 Fig LC-MS/MS chromatogram. LC-MS/MS chromatogram of the standard hormone solutions showing the E1, E2, E3 and Progesterone peaks.
(TIF)

S2 Fig Chromatographic LC/MS analysis. Chromatographic LC/MS analysis of the 3D printed constructs with control, E1, E2, E3 and Progesterone hormones.
(TIF)

S1 File Supplementary raw data. (XLSX)

## References

[1] Banks J. Adding value in additive manufacturing: Researchers in the United Kingdom and Europe look to 3D printing for customization. IEEE Pulse. 2013;4(6):22–6. doi: 10.1109/MPUL.2013.2279617 24233187 10.1109/MPUL.2013.2279617

[2] Mertz L. Dream it, design it, print it in 3-D: What can 3-D printing do for you? IEEE Pulse. 2013;4(6):15–21. doi: 10.1109/MPUL.2013.2279616 24233186 10.1109/MPUL.2013.2279616

[3] Gross BC, Erkal JL, Lockwood SY, Chen C, Spence DM. Evaluation of 3D printing and its potential impact on biotechnology and the chemical sciences. Anal Chem. 2014;86(7):3240–53. doi: 10.1021/ac403397r 24432804 10.1021/ac403397r

[4] Cui X, Boland T, D’Lima DD, Lotz MK. Thermal inkjet printing in tissue engineering and regenerative medicine. Recent Pat Drug Deliv Formul. 2012;6(2):149–55. 22436025 10.2174/187221112800672949 PMC3565591

[5] Duan B, Hockaday LA, Kang KH, Butcher JT. 3D bioprinting of heterogeneous aortic valve conduits with alginate/gelatin hydrogels. J Biomed Mater Res A. 2013 5;101(5):1255–64. doi: 10.1002/jbm.a.34420 23015540 10.1002/jbm.a.34420 PMC3694360

[6] Kailasam C, Cahill D. Review of the safety, efficacy and patient acceptability of the levonorgestrel-releasing intrauterine system. Patient Prefer Adherence. 2008 1;2:293–302. 19920976 10.2147/ppa.s3464 PMC2770406

[7] Programme UND, Fund UNP, Organization WH, Bank W, Research SP of, Reproduction D and RT in H. Long-term reversible contraception. Contraception. Elsevier; 2017 1 19;56(6):341–52.

[8] Jelovsek JE, Maher C, Barber MD. Pelvic organ prolapse. Lancet. 2007 p. 1027–38. 10.1016/S0140-6736(07)60462-0 17382829

[9] Roehl B, Buchanan EM. Urinary incontinence evaluation and the utility of pessaries in older women. Care Manag J. 2006 1;7(4):213–7. 17194058 10.1891/cmj-v7i4a007

[10] Weisman JA, Nicholson JC, Tappa K, Jammalamadaka U, Wilson CG, Mills DK. Antibiotic and chemotherapeutic enhanced three-dimensional printer filaments and constructs for biomedical applications. Int J Nanomedicine. 2015 1;10:357–70. doi: 10.2147/IJN.S74811 25624758 10.2147/IJN.S74811 PMC4296964

[11] Espey E, Ogburn T. Long-term reversible contraceptives: intrauterine devices and the contraceptive implant. Obstetrics and gynecology. 2011 3;117(3):705–719. doi: 10.1097/AOG.0b013e31820ce2f0 21343774 10.1097/AOG.0b013e31820ce2f0

[12] Edelman D, Berger G, Keith L. Intrauterine Devices and their complications. Dordrechr: Springer Netherlands; 1979. 263 p.

[13] Weisman JA, Jammalamadaka U, Tappa K, Nicholson JC, Ballard DH, Wilson CG, et al 3D printing antibiotic and chemotherapeutic eluting catheters and constructs. J Vasc Interv Radiol. Elsevier; 2015 2 2;26(2):S12.

[14] Ballard DH, Weisman JA, Jammalamadaka U, Tappa K, Alexander JS, Griffen FD. Three-dimensional printing of bioactive hernia meshes: In vitro proof of principle. Surgery. 2017 6;161(6):1479–81. doi: 10.1016/j.surg.2016.08.033 27726915 10.1016/j.surg.2016.08.033

[15] Tudela F, Kelley R, Ascher-Walsh C, Stone JL. Low Cost 3D Printing for the Creation of Cervical Cerclage Pessary Used to Prevent Preterm Birth. Obstet Gynecol. 2016 5;127:154S.

[16] Hakim J, Oluyemisi A, Buskmiller C, Krishnamurthy R, Cohn W, Dietrich JE. Innovative Use of 3D Printers in Gynecology. J Pediatr Adolesc Gynecol. Elsevier; 2015 4;28(2):e67.

[17] Cunha JAM, Mellis K, Sethi R, Siauw T, Sudhyadhom A, Garg A, et al Evaluation of PC-ISO for customized, 3D Printed, gynecologic 192-Ir HDR brachytherapy applicators. J Appl Clin Med Phys. 2015 1 8;16(1):5168 doi: 10.1120/jacmp.v16i1.5168 25679174 10.1120/jacmp.v16i1.5168 PMC5689973

[18] Ricotti R, Vavassori A, Bazani A, Ciardo D, Pansini F, Spoto R, et al 3D-printed applicators for high dose rate brachytherapy: Dosimetric assessment at different infill percentage. Phys Medica. 2016 12 1;32(12):1698–706. 10.1016/j.ejmp.2016.08.016 27592531

[19] Sethi R, Cunha A, Mellis K, Siauw T, Diederich C, Pouliot J, et al Clinical applications of custom-made vaginal cylinders constructed using three-dimensional printing technology. J Contemp Brachytherapy. Termedia Publishing; 2016 6;8(3):208–14. doi: 10.5114/jcb.2016.60679 27504130 10.5114/jcb.2016.60679 PMC4965501

[20] Laronda MM, Rutz AL, Xiao S, Whelan KA, Duncan FE, Roth EW, et al A bioprosthetic ovary created using 3D printed microporous scaffolds restores ovarian function in sterilized mice. Nature Communications. 2017 5 16;8:15261 doi: 10.1038/ncomms15261 28509899 10.1038/ncomms15261 PMC5440811

