# Supplementary figures and images for: Medication eluting devices for the field of OBGYN (MEDOBGYN): 3D printed biodegradable hormone eluting constructs, a proof of concept study
Source: PLoS One. 2017 Aug 10;12(8):e0182929. doi: 10.1371/journal.pone.0182929 (PMC5552136; doi:10.1371/journal.pone.0182929)

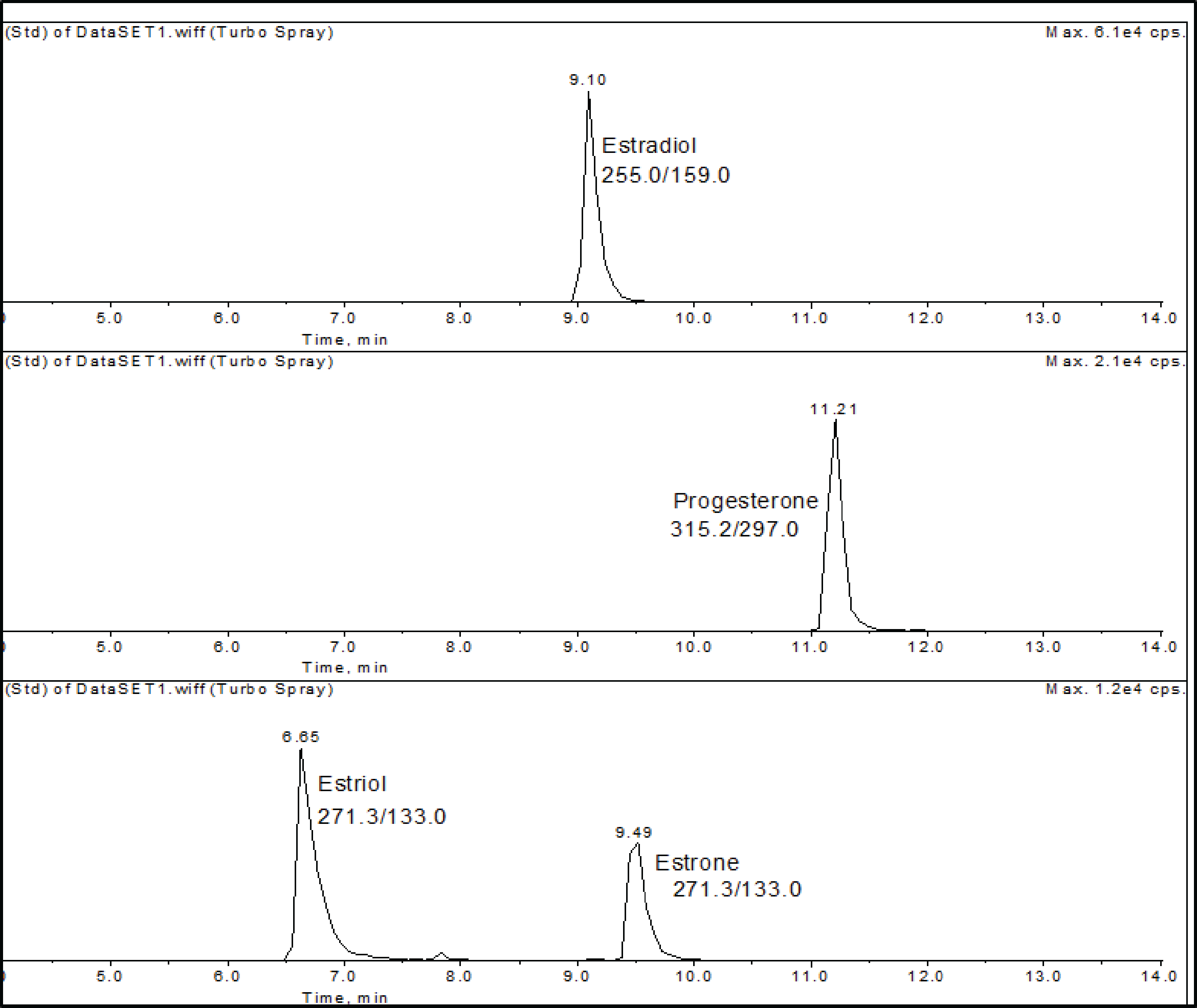

Supplement: S1 Fig — LC-MS/MS chromatogram of the standard hormone solutions showing the E1, E2, E3 and Progesterone peaks. (TIF) [file pone.0182929.s001.tif]

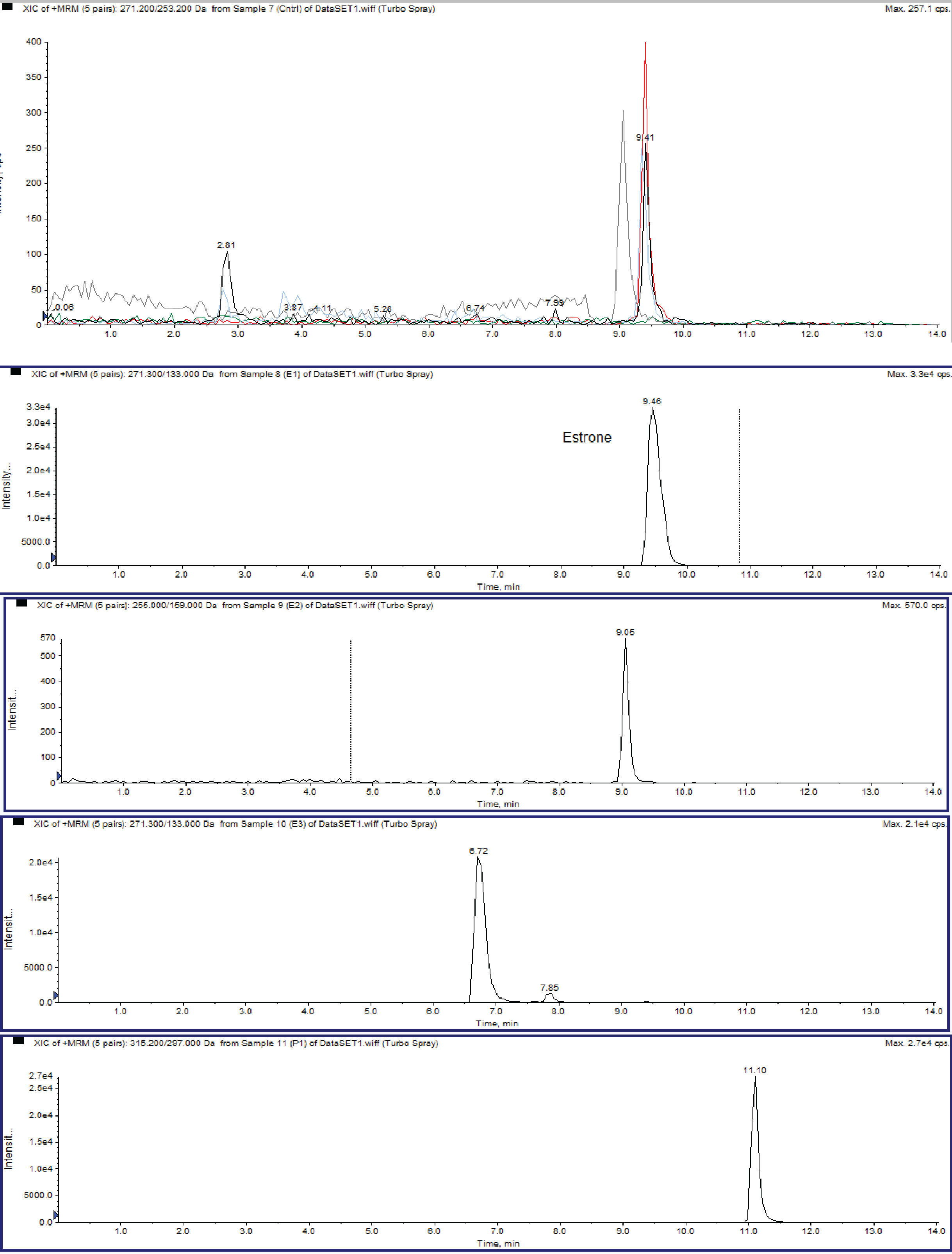

Supplement: S2 Fig — Chromatographic LC/MS analysis of the 3D printed constructs with control, E1, E2, E3 and Progesterone hormones. (TIF) [file pone.0182929.s002.tif]
